# Proteomic analysis of 92 circulating proteins and their effects in cardiometabolic diseases

**DOI:** 10.1186/s12014-023-09421-0

**Published:** 2023-08-07

**Authors:** Corinne Carland, Grace Png, Anders Malarstig, Pik Fang Kho, Stefan Gustafsson, Karl Michaelsson, Lars Lind, Emmanouil Tsafantakis, Maria Karaleftheri, George Dedoussis, Anna Ramisch, Erin Macdonald-Dunlop, Lucija Klaric, Peter K. Joshi, Yan Chen, Hanna M. Björck, Per Eriksson, Julia Carrasco-Zanini, Eleanor Wheeler, Karsten Suhre, Arthur Gilly, Eleftheria Zeggini, Ana Viñuela, Emmanouil T. Dermitzakis, James F. Wilson, Claudia Langenberg, Gaurav Thareja, Anna Halama, Frank Schmidt, SCALLOP Consortium, Daniela Zanetti, Themistocles Assimes

**Affiliations:** 1 Department of Medicine, University of Pennsylvania, Philadelphia, PA USA; 2 Institute of Translational Genomics, Helmholtz Zentrum München – German Research Center for Environmental Health, Neuherberg, Germany; 3 Department of Medical Epidemiology and Biostatistics, Karolinska Institute, Stockholm, Sweden; 4 Pfizer Worldwide Research, Development and Medical, Stockholm, Sweden; 5 Department of Medicine, Division of Cardiovascular Medicine, Stanford University School of Medicine, Stanford Cardiovascular Institute, Palo Alto, CA USA; 6 Department of Medical Sciences, Clinical Epidemiology, Uppsala University, Uppsala, Sweden; 7 Department of Surgical Sciences, Medical Epidemiology, Uppsala University, Uppsala, Sweden; 8 Anogia Medical Centre, Anogia, Greece; 9 Echinos Medical Centre, Echinos, Greece; 10 Department of Nutrition and Dietetics, School of Health Science and Education, Harokopio University of Athens, Athens, Greece; 11 Department of Genetic Medicine and Development, Faculty of Medicine, University of Geneva Medical School, Geneva, Switzerland; 12 Centre for Global Health Research, Usher Institute, University of Edinburgh, Edinburgh, Scotland; 13 MRC Human Genetics Unit, Institute of Genetics and Molecular Medicine, University of Edinburgh, Edinburgh, Scotland; 14 Department of Medical Epidemiology and Biostatistics, Karolinska Institute, Stockholm, Sweden; 15 Cardiovascular Medicine, Medicine, Karolinska Institute, Stockholm, Sweden; 16 MRC Epidemiology Unit, University of Cambridge, Cambridge, UK; 17 Bioinformatics Core, Cornell Medicine - Qatar Research, Doha, Qatar; 18 Technical University of Munich (TUM) and Klinikum Rechts der Isar, TUM School of Medicine, Munich, Germany; 19 Biosciences Institute, Faculty of Medical Sciences, University of Newcastle, Newcastle, UK; 20 Computational medicine, Berlin Institute of Health at Charité - Universitätsmedizin Berlin, Berlin, Germany; 21 Precision Healthcare University Research Institute, Queen Mary University of London, London, UK; 22 Proteomics Core, Research, Weill Cornell Medicine - Qatar, Doha, Qatar

**Keywords:** Proteomics, Cardiology, Genomics, Mendelian randomization, GWAS, Sex heterogeneity

## Abstract

**Background:**

Human plasma contains a wide variety of circulating proteins. These proteins can be important clinical biomarkers in disease and also possible drug targets. Large scale genomics studies of circulating proteins can identify genetic variants that lead to relative protein abundance.

**Methods:**

We conducted a meta-analysis on genome-wide association studies of autosomal chromosomes in 22,997 individuals of primarily European ancestry across 12 cohorts to identify protein quantitative trait loci (pQTL) for 92 cardiometabolic associated plasma proteins.

**Results:**

We identified 503 (337 cis and 166 trans) conditionally independent pQTLs, including several novel variants not reported in the literature. We conducted a sex-stratified analysis and found that 118 (23.5%) of pQTLs demonstrated heterogeneity between sexes. The direction of effect was preserved but there were differences in effect size and significance. Additionally, we annotate trans-pQTLs with nearest genes and report plausible biological relationships. Using Mendelian randomization, we identified causal associations for 18 proteins across 19 phenotypes, of which 10 have additional genetic colocalization evidence. We highlight proteins associated with a constellation of cardiometabolic traits including angiopoietin-related protein 7 (ANGPTL7) and Semaphorin 3F (SEMA3F).

**Conclusion:**

Through large-scale analysis of protein quantitative trait loci, we provide a comprehensive overview of common variants associated with plasma proteins. We highlight possible biological relationships which may serve as a basis for further investigation into possible causal roles in cardiometabolic diseases.

**Supplementary Information:**

The online version contains supplementary material available at 10.1186/s12014-023-09421-0.

## Background

Human plasma contains many circulating proteins that are derived from multiple organs and that participate in a wide range of biological processes. These proteins may be secreted directly into circulation or may spill over into the blood from their organs of origin. Clinically, circulating proteins can be used as biomarkers (e.g. N-terminal pro-brain natriuretic peptide in congestive heart failure [1]) and also as drug targets (e.g. proprotein convertase subtilisin/kexin type 9 serine protease (PCSK9) in hypercholesterolemia [2]). Drug targets with human genetics evidence behind them are twice as likely to lead to approved drugs [3], with 66% of FDA-approved drugs having prior generated genetics evidence [4]. Further, understanding patterns of protein dysregulation in disease can offer insights into pathophysiology. Cardiometabolic diseases are particularly important to study as they represent the leading cause of death globally and continue to rise in incidence [5, 6].

Genome wide association studies (GWAS) can be used to evaluate the genetic underpinnings of protein abundance. Specifically, protein quantitative trait loci (pQTLs) are genetic loci that are found to be associated with protein levels. Recent technological advances have allowed for the high throughput quantification of protein levels in plasma samples [7]. This development has facilitated several large-scale proteomics studies of plasma, which have provided insight into the genetic underpinnings of circulating proteins and illuminated potential novel therapeutic targets [8–10].

In this work, we present the results of a genome-wide pQTL meta-analysis of 12 European cohorts with measurements of 90 circulating proteins selected for being involved in key metabolic processes including cellular metabolic processes, cell surface receptor signaling pathways, regulation of phosphorylation, and cell adhesion. We use colocalization and Mendelian randomization (MR) methods to find evidence for potentially causal relationships between proteins and diseases. Further, we conduct a sex-stratified meta-analysis to shed light on differences in the magnitude of genetic associations between males and females.

## Methods

### Protein quantification assay

We used the Proximity Extension Assay (PEA) technology [11] to measure 92 proteins on the Olink Target Metabolism (Uppsala, Sweden), one of 14 carefully designed panels for relative quantification of proteins in humans (Additional file 1: Table S1). The assays in this panel were carefully selected to include proteins involved in key biological processes such as cellular metabolic processes, cell surface receptor signaling pathways, regulation of phosphorylation and cell adhesion. The PEA technology includes a pair of oligonucleotide-labeled antibody probes that bind independently to a target protein in a sample close enough to allow the two oligonucleotides to hybridize. DNA polymerase in PCR then amplifies these unique “barcode” DNA which are subsequently quantified with qPCR. Olink quantification levels below the level of detection were included.

### Cohorts

Investigators from 12 primarily European ancestry cohorts with both genetic data and protein measurements of the metabolism panel provided data for this study. A detailed description of all included cohorts including design, inclusion/exclusion criteria, sample size, and genetic array used is included in Additional file 1: Table S2. Each cohort imputed their genetic array data to the 1000 Genomes Project phase 3 reference or later or to the Haplotype Reference Consortium (HRC), except for the MANOLIS and Pomak cohorts, which underwent whole-genome sequencing. Two cohorts included only participants from a single sex: males in the Uppsala Longitudinal Study of Adult Men (ULSAM) and females in the Swedish Mammography Cohort—Clinical (SMCC).

### Genome-wide association analysis

Each cohort performed a GWAS on measured circulating protein levels for each protein. Genetic variant information was filtered out using the following criteria: missing calls > 3%, Hardy Weinberg Equilibrium (HWE) P < 5 × 10^–6^, minor allele frequency < 0.01. The relative protein abundances were then rank-based inverse-normal transformed before the GWAS was performed adjusted for age, sex, storage time, plate, and the first 10 principal components. Additional details of each GWAS are provided in Additional file 1: Table S2. All other cohorts with both men and women performed sex stratified analyses in addition to pooled analyses.

### GWAS data cleaning and meta-analysis

GWAS summary statistics for a given protein were excluded entirely if greater than 80% of sample measurements were below Olink’s limit of detection (Additional file 1: Table S3). All summary statistics also underwent quality control using EasyQC [12]. Variants were excluded if minor allele count was less than or equal to 20, imputation quality was less than 0.4, or a variant was monomorphic. Sex chromosomes analyses were also excluded.

Proteins were included for meta-analysis if there were at least three cohorts present after filtering. Meta-analysis was performed using a random effects model implemented in Genome Wide Association Meta-Analysis (GWAMA version 2.2.2) [13] to account for potential heterogeneity of associations across cohorts. Meta-analysis results were conducted in duplicate at two different research centers using two different pipelines and then compared to ensure concordance. Study wide significance was defined at a Bonferroni corrected value of P < 5.6 × 10^–10^ (genome wide significance 5 × 10^–8^ divided by 90 proteins).

### Selection of independent variants

We excluded genetic variants present in less than two cohorts. We identify independent pQTLs through two methods: clumping and conditional analysis. Clumping for independent variants was performed with Plink [14] through the clump option with parameters -clump-r^2^ set to 0 and -clump-kb set to 500 kB. A subset (n = 11,227) of individual level data from the Human Reference Consortium were used as reference [15, 16].

We then conducted conditional-joint analysis in GCTA using the -cojo-slct option [17, 18] combined with the Haplotype Reference Consortium (HRC release 1.1; EGAD00001002729) panel as the LD reference, requiring a GWAS P < 5.6e−10 and a COJO conditional P < 5e−5 for a SNP to be selected. Meta-analysis summary data were filtered for MAF > 0.01 and r^2^ > 0.05 to minimize the probability that additional signals were driven by linkage disequilibrium (LD) with the primary signal. We also explored if all pQTLs or their SNPs in LD (r^2^ > 0.3) were associated with their eGenes in GTEx v8 database using the LDexpress tool [19].

Cis pQTLs were defined as a signal within 0.5 Mb of the gene encoding the protein. All other signals were defined as trans.

### Comparison of pQTLs to prior published data

We compared our pQTL results to the recently released summary statistics of the UK Biobank Pharma Proteomics Project (UKB-PPP) which measured 1,463 proteins in 54,206 participants including all of the proteins on the Olink Target Metabolism panel [20]. We examine pQTLs overlap of our pQTLs in the UKB-PPP (Additional file 1: Table S13a) and also the overlap of the UKB-PPP pQTLs in our study (Additional file 1: Table S13b). We found that 475 out of our 503 pQTLs (94.4%) overlapped with UKB-PPP. Among the 475 pQTLs, 454 (95.6%) and 462 (97.3%) were replicated at P < 1.05e−4 (accounting for 475 pQTLs tested) and nominal P < 0.05, respectively. While assessing the replication of UKB-PPP pQTLs in our study, we found that 488 pQTLs for 87 Metabolism proteins overlapped with our study. Among these 488 pQTLs, 204 (41.8%) and 388 (79.5%) pQTLs were replicated in our study at P < 1.02e−4 (accounting for 488 pQTLs tested) and nominal P < 0.05, respectively.

### Meta-analysis of sex stratified GWAS

The 12 cohorts included a total of 10,885 women and 12,112 men. We conducted two different types of meta-analyses with the sex stratified GWAS. First, we conducted a meta-analysis analysis, using a random effects model implemented in GWAMA. We then conducted a second meta-analysis segregating by sex. This resulted in male and female specific pQTLs. Second, we used the -sex option of the GWAMA software to perform a sex stratified meta-analysis and to highlight the heterogeneity between sexes [21]. We ran heterogeneity tests of all the significant 503 pQTLs detected in the sex stratified GWAS. The heterogeneity significance threshold for multiple testing was set to 9.9 × 10^–5^ (0.05/503).

### Phenotypic and genetic correlation

We calculated pairwise Pearson correlation coefficients (*r*) for all 90 proteins using the R software (version 3.3.2). We also applied LD-score regression (LDSC) [22] to estimate the heritability (h^2^) of each protein and to quantify pair-wise genetic correlations between proteins. For these analyses, we used pre-calculated LD scores for Europeans in HapMap Phase 3 [23]. The phenotypic and genetic correlation matrices were individually ordered using a hierarchical clustering approach (Additional file 1: Tables S7 and S8; Additional file 2: Figures S4 and S5).

### Mendelian randomization

Two sample MR was performed to assess potentially causal effects of proteins on a wide range of diseases (Additional file 1: Table S4) [24] using the R package *TwoSampleMR* [25]. To identify independent genetic instruments with a low probability of pleiotropy for these analyses, we filtered all detected pQTLs at linkage disequilibrium (LD) r^2^ threshold of 0.01 and removed pQTLs in known pleiotropic regions, including those in the MHC region, *ABO*, *CFH*, and *VTN* gene coding regions. Among remaining SNPs, we created two sets of instruments: (1) cis pQTLs with P < 5 × 10^–8^ and (2) cis pQTLs with P < 5 × 10^–8^ plus trans pQTLs with P < 5.6 × 10^–10^. While trans instruments may be more prone to pleiotropy, they have value in MR analysis by increasing variance explained by the tested protein. Additionally, they may represent an upstream mechanism of action. For single instruments, we generated an instrument variable (IV) Wald ratio estimate while summary IV estimates for multiple instruments were generated by through an inverse variance weighted fixed effect meta-analysis of individual instruments. A Benjamini–Hochberg FDR < 0.05, assigned separately in cis pQTLs and cis plus trans pQTLs, was used as a threshold of significance for a significant MR result.

### Colocalization

For all 18 proteins with a significant protein-disease association in the two-sample Mendelian randomization analysis, genetic colocalization was carried out with 15 selected unique traits of cardiometabolic relevance (Additional file 1: Table S11). Colocalization was performed using the coloc.fast function from the gtx R package [26]. The method is equivalent to coloc by Giambartolomei et al. [27] and assumes one causal variant at each associated locus. To satisfy this assumption at loci with more than one independent variant, each independent variant was conditioned on all other independent variants at the locus using the –cojo–cond function implemented in GCTA version 1.93.0, using the HRC panel (release 1.1; EGAD00001002729) as an LD reference; each independent variant was tested individually using the resulting summary statistics as input. We define positive colocalization as a posterior probability 4 (PP4) of more than 80%, as in the original coloc paper. We performed additional analysis with all available traits in PhenoScanner [28, 29] extracting summary statistics for regions ± 1 Mb of the independent pQTL. Full results for colocalization are provided in Additional file 1: Table S11.

## Results

### GWAS meta-analysis and pQTL discovery

**Fig. 1:**
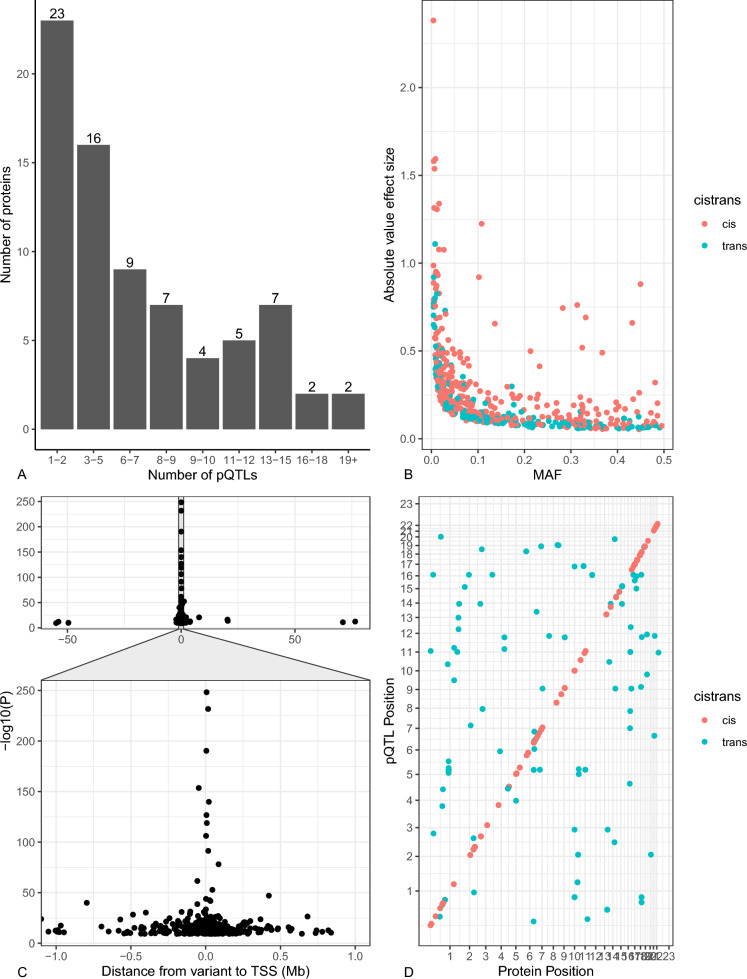
**a** Distribution of the number of pQTLs per protein. We observe a range of 1–2 pQTLs per protein for 23 proteins and 19 + pQTLs for each of two proteins. **b** Minor allele frequency (MAF) versus absolute value of effect size. As the MAF increases, size of the effect tends to increase. **c**. Distance from variant to transcription start site of protein (TSS) versus negative log P. Variants plotted here include all SNPs on the same chromosome as the coding region of the protein. The most statistically significant pQTLs are closest to the TSS. **d** pQTL position versus location of protein coding gene

A total of 90 proteins in up to 22,997 individuals from 12 cohorts passed quality-control criteria and were included in the GWAS meta-analysis. The meta-analysis identified pQTLs for plasma levels of 77 proteins (Additional file 1: Table S5). We found 178 independent pQTLs at a Bonferroni-corrected significance of P < 5.6 × 10^–10^. After conditional analysis, we found an additional 325 conditionally independent pQTLs at P < 5.6 × 10^–10^. Thus, we identified 503 (337 cis and 166 trans) independent pQTLs in total (Fig. 1, Additional file 1: Table S5).

Most proteins were associated with five or fewer pQTLs, and 18 proteins had greater than 10 conditionally-independent pQTLs. Among these 18 proteins, we observed substantial variability in the distribution of cis- and trans-pQTLs. For example, the T-cell surface glycoprotein CD1c (CD1C) has 12 trans- and only one cis-pQTL. Eight of its trans-pQTLs are located within the human major histocompatibility complex (MHC) region (chr6:29691116–33054976); CD1c and MHC molecules have similar functions in T-cell immune responses [30], and our findings point to complex interplay between them. Conversely, meprin A subunit beta (MEP1B) has 22 cis- and only one trans-pQTL, suggesting complex local genetic regulation of circulating MEP1B at the encoding gene region. Additionally, 27.24% (137/503) of all pQTLs or their SNPs in LD (r^2^ > 0.3) also demonstrated associations with their eGenes in GTEx v8 database at P < 1 × 10^–4^, providing some evidence that pQTL discovery may be a downstream consequence of effects of the same genetic variant on gene expression (Additional file 1: Table S6).

### Sex stratified meta-analysis

**Fig. 2:**
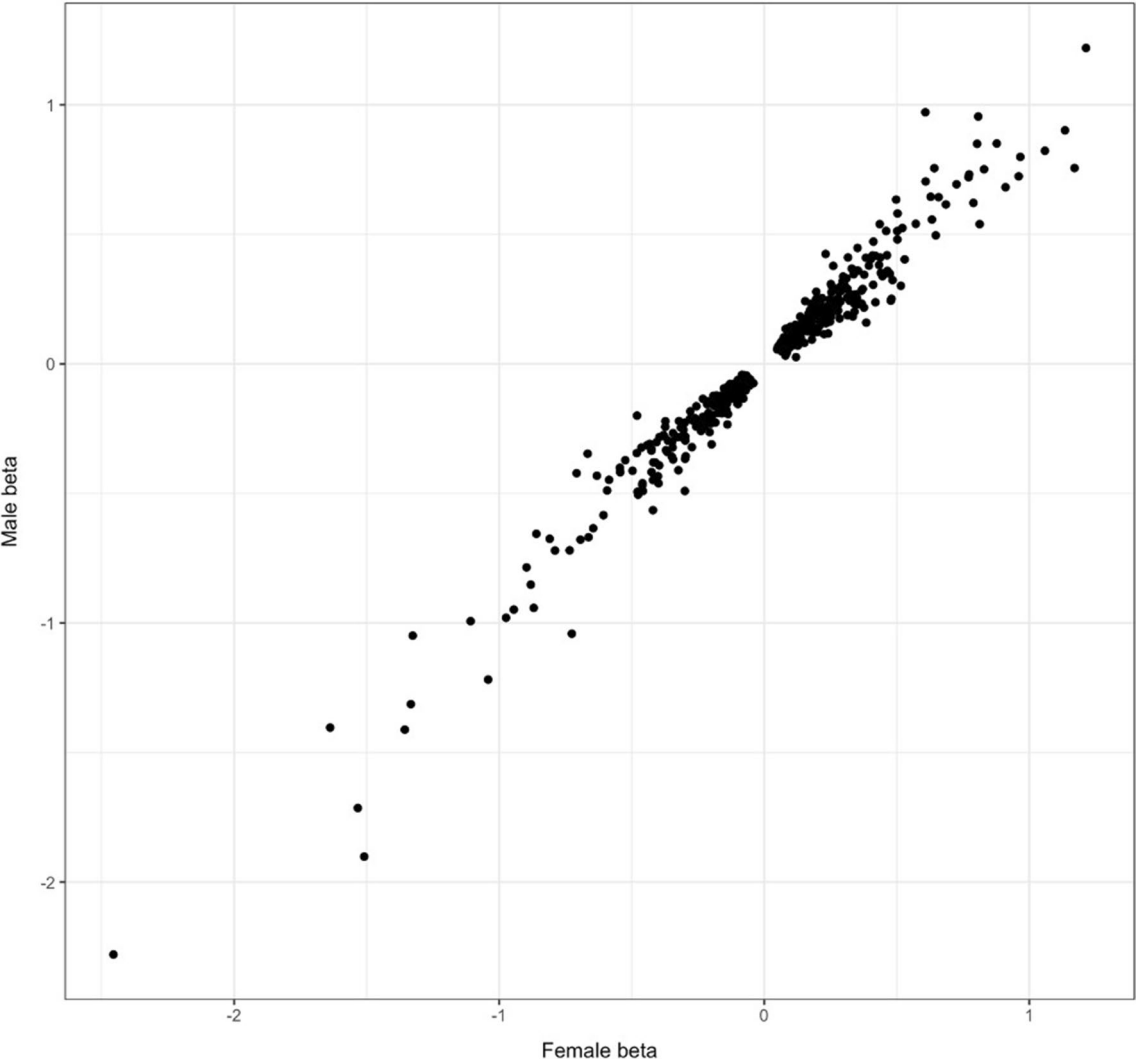
pQTL betas in males versus females for pQTLs significant in both sexes

We identified 258 pQTLs among men (at P < 5 × 10^–8^; 130 at a Bonferroni-corrected significance threshold of P < 5.6 × 10^–10^) and 552 pQTLs among women (at P < 5 × 10^–8^; 399 at P < 5.6 × 10^–10^). This sex-stratified meta-analysis confirmed a concordance in the direction of effects between males and females for all 503 pQTLs discovered in the pooled meta-analysis (Fig. 2). All independent pQTLs from the pooled meta-analysis were tested for sex specific heterogeneity (Additional file 1: Table S14). Using a significance threshold corrected for multiple testing (P < 9.9 × 10^–5^), 118 (23.5%) pQTLs demonstrated heterogeneity between sexes. Of these, 97 (82.2%) pQTLs had greater absolute beta values in females, versus 21 (17.8%) in males; one cis-pQTL for adenosylhomocysteinase (AHCY; rs34563588) in particular, was significant only in males. Sex heterogeneity was most significant for a cathepsin H (CTSH)-increasing pQTL (rs77362013; P = 1.35 × 10^–220^; beta_male_ = 0.616; beta_female_ = 0.685).

**Fig. 3:**
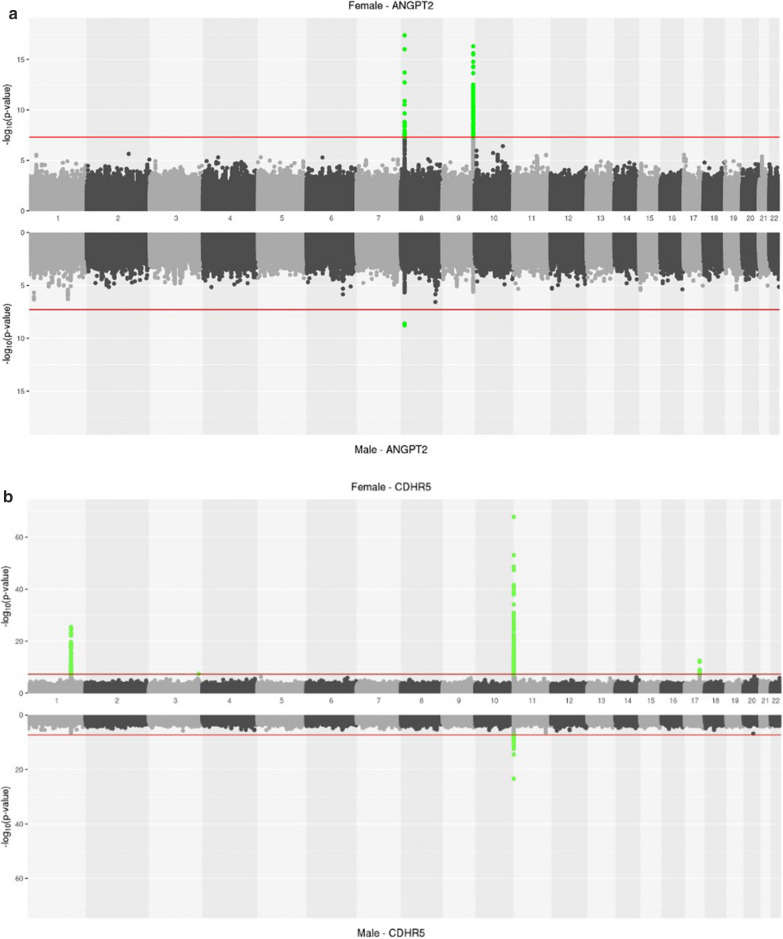
**a** Miami plot for ANGPT2. Two loci are seen for females (chromosomes 8 and 9) but in males, there is only a significant SNP on chromosome 8. **b** Miami plot for CDHR5. Females demonstrate significant associations on chromosomes 1, 11, and 17 while males have a significant locus on chromosome 11

The heterogeneity drove differences in signal detection between males and females for some proteins. For example, for CDHR5, females have significant associations in chromosomes 1 (rs12134610, trans), 11 (rs117818025, cis), and 17 (rs1801689, trans); while males only have a significant association in chromosome 11 (rs12804878, cis). Similarly, we detect two loci in females for angiopoietin 2 (ANGPT2): one in chromosome 8 (rs13264652, cis) and one in chromosome 9 (rs9411492, trans). We observe replication in males for the cis-pQTL, but not the trans-pQTL (Fig. 3). The trans*-*pQTL is strongly associated with *ABO* expression in the thyroid (GTEx), where *ANGPT2* is also highly expressed. Differences in thyroid function and prevalence of autoimmune thyroid disease between males and females [31] could drive this female-specific effect. Four additional proteins have significant pQTLs in the sex stratified analysis that were attenuated in the combined meta-analysis: GRAP2 (rs79376201) and KYAT1 (rs3750319) in females; and CRKL (rs188792857) and SNAP23 (rs150285625 and rs188792857) in males.

### Phenotypic and genotypic correlation

We detected overall phenotypic and genotypic correlation across the proteins analyzed (Additional file 1: Tables S7 and S8; Additional file 2: Figures S3 and S4). The highest positive phenotypic correlation detected was between the synaptosome associated protein 23 (SNAP23) and the disabled homolog 2 (DAB2) protein (*r* = 0.930). The highest negative phenotypic correlation detected was between the amyloid beta precursor like protein 1 (APLP1) and the heparin binding growth factor (HDGF) (*r* = − 0.377). The highest positive genetic correlation detected was between the CXADR-like membrane protein (CLMP) and the FAM3 metabolism regulating signaling molecule C (FAM3C) protein (*r* = 0.821). The highest negative genetic correlation detected was between the BAG cochaperone 6 (BAG6) protein and the protein phosphatase 1 regulatory inhibitor subunit 2 (PPP1R2) (*r* = − 0.336). In general, we observed that high genotypic correlation does not always translate to strong phenotypic correlation and vice versa (Additional file 1: Table S9).

The heritability of the proteins studied was within the range of 0 ≤ h^2^_g_ ≤ 0.118 (median = 0.053; interquartile range = 0.047; Additional file 1: Table S10), although we note that accurate heritability estimation would require larger sample sizes.

### Two-sample Mendelian randomization and colocalization analysis

**Fig. 4:**
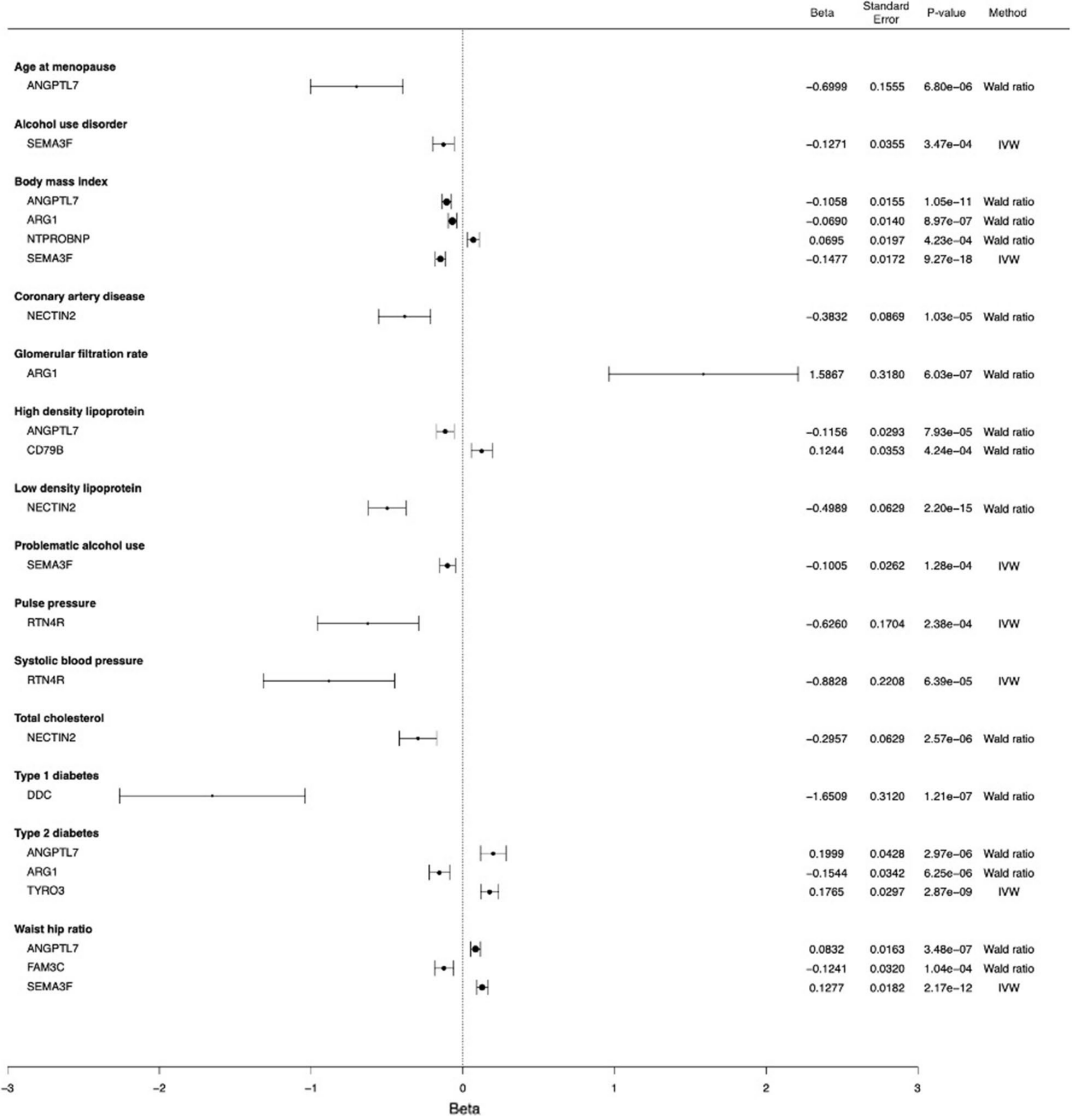
Forest plot of MR results using cis instruments

**Fig. 5:**
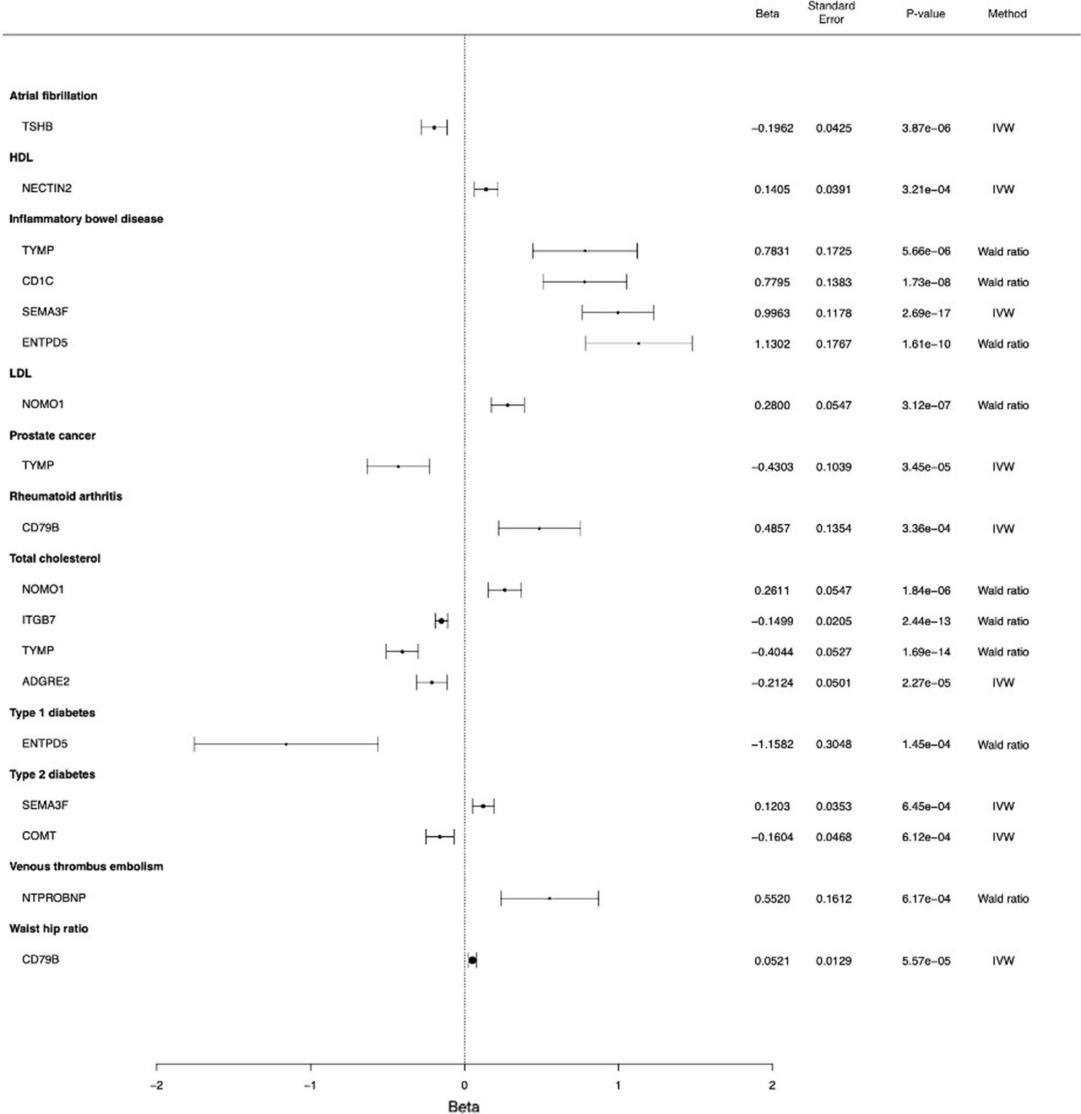
Forest plot of MR results using both cis and trans instruments. *IVW* inverse variance weighted

We find causal associations for 18 proteins and a total of 19 phenotypes/disease using cis and cis-plus-trans instruments (Figs. 4, 5, Table 1). Of the 18 proteins, 10 have associations using cis-only instruments. Ten proteins (ANGPTL7, SEMA3F, ARG1, NTPROBNP, NECTIN2, CD79B, RTN4R, ENTPD5, TYMP, NOMO1) are associated with more than one outcome (Table 1). We note that two-sample MR relies on specific assumptions [32] that can lead to false positives when violated; to strengthen our findings, we performed additional colocalization analysis for the 18 proteins and their associated (and other relevant) traits (Additional file 1: Table S11 and S12). We observe positive colocalization [posterior probability (PP) > 80%] for 5 of 18 proteins with the same traits (ADGRE2 and total cholesterol [TC]; ANGPTL7 and BMI, waist-hip ratio; ITGB7 and TC; NOMO1 and LDL, TC; SEMA3F and BMI, alcohol use), supporting the two-sample MR results.

**Table 1:** MR associations

Protein	Function	Association
ANGPTL7	Angiopoietin-related protein 7: formation and organization of extracellular matrix. Negative regulator of angiogenesis in cornea. Plays role in maintaining corneal avascularity and transparency	↓Age at menopause, ↓BMI, ↓HDL, ↑Type II diabetes, ↑WHR
SEMA3F	Semaphorin-3F: involved in cell signaling	↓Alcohol use disorder, ↓BMI, ↓Problematic alcohol use, ↑WHR, **↑Inflammatory bowel disease, ↑Type II diabetes**
ARG1	Arginase-1: enzyme in urea cycle converting L-arginine to urea and L-ornithine	↓BMI, ↑GFR, ↓Type II Diabetes
NTPROBNP	Natriuretic peptides B: hormone plays a role in mediating extracellular fluid volume and cardio-renal homeostasis	↑BMI, **↑VTE**
NECTIN2	Nectin-2: modulator of T-cell signaling	↓CAD, ↓LDL, ↓Total cholesterol, **↑HDL**
CD79B	B-cell antigen receptor complex-associated protein beta chain: involved in signal cascade activated by B-cell antigen receptor complex	↑HDL, **↑Rheumatoid arthritis, ↑WHR**
RTN4R	Reticulon-4 receptor: receptor for several ligands	↓Pulse pressure, ↓Systolic blood pressure
DDC	Aromatic-l-amino-acid decarboxylase: enzyme that catalyzes the conversion of l-3,4-dihydroxyphenylalanine (DOPA) to dopamine, l-5-hydroxytryptophan to serotonin and l-tryptophan to tryptamine	↓Type I diabetes
TYRO3	Tyrosine-protein kinase receptor: receptor tyrosine kinase that transduces signal from extracellular matrix to cytoplasm, binds several different ligands	↑Type II diabetes
FAM3C	Family with sequence similarity 3 member C: possible involvement in retinal laminar formation. Promotes epithelial to mesenchymal transition	↓WHR
TSHB	Thyrotropin subunit beta: subunit of hormone mediating thyroid function	**↓Atrial fibrillation**
ENTPD5	Ectonucleoside triphosphate diphosphohydrolase 5: a Uridine diphosphatase involved in protein N-glycosylation and ATP regulation	**↓Type 1 diabetes, ↑Inflammatory bowel disease**
CD1C	T-cell surface glycoprotein CD1c: protein presents to antigens to T-cell receptors on natural killer T-cells	**↑Inflammatory bowel disease**
TYMP	Thymidine phosphorylase: catalyzes the reversible phosphorolysis of thymidine	**↑Inflammatory bowel disease, ↑Total cholesterol, ↓Prostate cancer**
ITGB7	Integrin beta-7: adhesion molecule that mediates lymphocyte migration and homing to gut-associated lymphoid tissue	**↓Total cholesterol**
NOMO1	Nodal modulator 1: involved in membrane protein transport into the endoplasmic reticulum	**↑LDL, ↑Total cholesterol**
ADGRE2	Adhesion G protein-coupled receptor E2: cell surface receptor, promotes cell attachment, granulocyte chemotaxis, degranulation, and adhesion	**↓Total cholesterol**
COMT	Catechol O-methyltransferase: enzyme that catalyzes the O-methylation, and thereby the inactivation, of catecholamine neurotransmitters and catechol hormones	**↓Type II diabetes**

Each protein and the significant associations through Mendelian randomization. Up arrow indicates that an increased amount of the protein is associated with an increased or higher value of the outcome (e.g. increasing ANGPTL7 is associated with a decreased BMI). All instruments are cis except for those associations indicated by bold font

*BMI* body mass index, *HDL* high density lipoprotein, *LDL* low density lipoprotein, *VTE* venous thrombus embolism, *WHR* waist hip ratio

Semaphorin 3F (SEMA3F) and angiopoietin-related protein 7 (ANGPTL7) are associated with the most traits, at six and five associations, respectively. Specifically, increasing levels of SEMA3F is associated with lower alcohol use disorder, problematic alcohol use, body mass index (BMI) and with greater waist hip ratios, inflammatory bowel disease, and type 2 diabetes. Increasing levels of ANGPTL7 is associated with a lower age of menopause, BMI, and high-density lipoprotein (HDL); and greater type 2 diabetes and waist hip ratios (Table 1; Figs. 4 and 5).

## Discussion

### Principal findings

In this analysis, we conduct a genome wide association meta-analysis of 90 circulating proteins in up to 22,997 European individuals. Our principal findings are four-fold: (1) After multiple-testing correction (alpha = 0.05), we identify a total of 503 independent pQTLs for 77 proteins; (2) We detect phenotypic and genotypic correlation across the proteins tested; (3) We conduct a sex-stratified analysis that reveals concordance in effect direction between sexes but with some heterogeneity; (4) We annotate trans-pQTLs with nearest genes and report plausible biological relationships and (5) Using a two-sample MR approach, we find support for causal associations for a total of 18 proteins, of which 10 are supported by genetic colocalization.

### MR results and comparison with prior literature

Our MR results suggest several associations between protein and disease. We find increasing levels of SEMA3F associated with decreasing risk of alcohol use disorder and problematic alcohol use and increased waist-to-hip ratio. Increasing levels of SEMA3F is also associated with increased risk of inflammatory bowel disease and type 2 diabetes through trans instruments. In agreement with our findings, a previous GWAS found an association with a locus at a different class of semaphorin, SEMA3A, to be associated with decreased risk of alcohol dependence and major depression in African Americans [33]. The semaphorins are a set of secreted and membrane proteins that play an important role in axon development and neuronal connectivity [34].

We find that increased levels of angiopoietin-related protein 7 (ANGPTL7) are associated with decreased age at menopause, decreased HDL, increased risk for type 2 diabetes, and increased waist to hip ratio (corrected for BMI). This is supported by genetic colocalization of the cis pQTL with signals for BMI (PP4 = 91.9%) and waist to hip ratio (PP4 = 92.1%); and colocalization between two non-pleiotropic trans pQTLs for ANGPTL7 (rs10893498 and rs535064984) and signals for low-density lipoprotein (LDL) levels (PP4_rs10893498_ = 97.6%; PP4_rs535064984_ = 99.9%). In general, our results suggest that increased ANGPTL7 is associated with increasing risk of metabolic syndrome, with the exception of BMI, where increased ANGPTL7 is associated with decreased BMI. While our MR results suggest that increased protein levels are associated with decreased BMI, one small observational study finds the opposite result, where ANGPTL7 is increased in subjects with obesity [35]. Interrogation of the GWAS Catalog finds that there is an association between SNPs mapped to the ANGPTL7 gene and both BMI and intraocular pressure [36].

Additionally, we find an association of RTN4R with systolic blood pressure and pulse pressure. RTN4R, or reticulon-4 receptor, is a receptor subunit for RTN4 which is known for being a myelin-associated inhibitor of axon regeneration [37]. This association has not been previously reported and may suggest some vascular effects of this protein that are not yet understood. Replication of this finding in additional cohorts would be an important next step.

Finally, we replicate several clinically known associations. We highlight a protective role of increased levels of thyroid stimulating hormone subunit beta (TSHB) against atrial fibrillation. TSHB is released by the pituitary gland to stimulate thyroid production of triiodothyronine (T3) and thyroxine (T4). Generally, high TSH levels are an indication of low concentrations of thyroid hormones, or hypothyroidism. Correspondingly, we observe colocalization of a known trans pQTL for TSHB (rs7695810; MAF = 0.181; beta = − 0.105; SE = 0.012; P = 3.89 × 10^–18^) with signals for self-reported hypothyroidism (PP4 = 92.6%) and treatment for hypothyroidism (91%) [38]. Since the opposite condition, hyperthyroidism, is a known cause of atrial fibrillation [39], it is consistent that increased levels of TSHB would be inversely associated with the arrythmia. Furthermore, we identify several associations of autoimmune diseases with proteins in the immune pathway including inflammatory bowel disease with T-cell surface glycoprotein (CD1C) and rheumatoid arthritis with B-cell antigen receptor complex-associated protein beta chain (CD79B).

### Trans pQTL nearest gene annotation

The protein trans-pQTLs were annotated with information on nearest genes (Additional file 1: Table S12). Previous work has suggested that the gene nearest the lead variant is often the causal gene, although not always [40]. The Olink protein and the nearest gene for each trans-pQTL were text-mined to gain insights into potential connections between the gene and the protein. A trans-pQTL for plasma ghrelin (GHRL), rs2894342, is located ~ 2000 base pairs upstream of the *MLN* gene. *MLN* encodes motilin, which is expressed in the gastrointestinal tract and in the brain, and regulates interdigestive contractile activity of the gastrointestinal tract. The observed trans-pQTL for ghrelin suggests that genetic regulation of motilin directly influences plasma ghrelin concentrations, providing new evidence of directional regulation of these digestive proteins. Another protein measured in our study, neuronal pentraxin 2 receptor (NPTXR), was associated with a trans-pQTL located ~ 20 kb downstream of *NPTX2*, which encodes a ligand for the neuronal pentraxin 2 receptor. Both proteins are enriched for expression in the cerebral cortex [41] but our data suggest that the signaling pathway is likely to be active also in the circulation.

For plasma ANGPTL7, we observed 3 trans-pQTLs located near *MRC1, ST3GAL4,* and *ASGR2*. *MRC1* encodes the mannose receptor C-type 1, which is expressed in the lung and on Kupffer cells in the liver, where it mediates endocytosis of glycoproteins [41]; ASGR2 is also involved in endocytosis of plasma glycoproteins, specifically those in which the terminal sialic acid residue on their carbohydrate moieties has been removed; and ST3GAL4 is an enzyme catalyzing terminal sialylation of glycoproteins. Experimental validation will be needed to determine if ANGPTL7—which is a 45 kDa glycoprotein—is directly modulated by these respective post-translational actions.

### Sex-specific meta-analysis

We identify pQTLs both in pooled and sex-stratified cohorts. A heterogeneity analysis reveals that there was full concordance of the direction of effects of all reported pQTLs from the pooled meta-analysis; however, 23.5% of pQTLs demonstrated heterogeneity between sexes. Interestingly, a large majority of these pQTLs had greater effect sizes in females compared to males. The reason behind this is unclear, but one possibility is that this could be an effect of higher prevalence of cardiometabolic medication in males versus females [42], which may affect protein levels. A similar trend has been observed in a GWAS of body fat distribution, where the authors find a high degree of sex-heterogeneity, with almost 95% of the implicated variants exhibiting larger effects in females [43]. Other GWAS have found evidence for sex-specific associations in abdominal and visceral fat [44], renal cell carcinoma [45] and longevity [46]. Literature in heterogeneity between sexes and sex-specific differences in pQTLs are limited [47].

### Conclusions

The main strength of our analysis lies in the large sample sizes comprising multiple cohorts, which maximizes power to detect even lower-frequency variants of smaller effect sizes. We also present causal associations between protein and disease that are based on multiple inference method approaches, such as MR and colocalization analyses.

However, there are several limitations to our work. Firstly, the proteins tested were limited to those detectable in blood and available on Olink’s Metabolism panel. This means that detected pQTLs are not representative of all cell types or tissues, which limits interpretation of their biological roles. Secondly, MR associations may be confounded by pleiotropic genetic instruments and reverse causality [48]. To address and/or minimize the former, we excluded all pQTLs located in known pleiotropic regions (Methods) and performed additional MR analyses using only cis instruments (Fig. 4), although we note that this does not completely eliminate confounding. Thirdly, the participants included in the genetic analyses were of European ancestry only; hence, our results may not be generalizable to other ethnic groups.

Through a large-scale pQTL analysis, we provide a comprehensive overview of the low-frequency to common variant architecture of 90 proteins in the blood and describe their heritability and sex-specific differences. These serve as a starting point for further inquiry into possible causal roles in complex diseases that may complement case–control studies of proteomic biomarkers and other drug target validation efforts. Importantly, all results should be substantiated by orthogonal validation. Further future directions include rare variant analysis [49] and cell type and tissue-specific analysis, which will provide a more complete picture of the complex genetic architecture underlying proteins, allowing us to harness the full potential of pQTLs.

### Supplementary Information

**Additional file 1: Table S1.** Protein functions. **Table S2.** Cohort Information. **Table S3.** Protein assay information. **Table S4.** MR outcomes. **Table S5.** **a** Pooled pQTLs. **b** Female pQTLs. **c** Male pQTLs. **d** Additional pQTLs MR. **Table S6.** eQTL analysis. **Table S7. **Phenotypic correlation. **Table S8.** Genetic Correlation. **Table S9.** Correlations Comparison. **Table S10.** Heritability Results. **Table S11. a** Colocalization sum stats. **b** Colocalization phenoscanner. **c** Colocalization reference. **Table S12.** Trans pQTL annotation. **Table S13**. **a** pQTLs replication. **b** External pQTLs lookup. **Table S14.** Sex heterogeneity pQTLs.

**Additional file 2: Figure S1.** Steps of analysis performed, as described in methods section. **Figure S2.** Meta-analysis methods and determination of primary and secondary pQTLs and Mendelian randomization instruments. **Figure S3.** Phenotypic correlation matrix across all proteins analyzed. **Figure S4.** Genotypic correlation matrix across all proteins analyzed.

## Data Availability

The genome-wide association study meta-analysis of all plasma protein levels will be available on Dryad. Individual level proteomic data will not be shared in public databases as consent for such sharing has not been uniformly obtained from participants of these studies. Controlled access to individual data may be available to qualified investigators for some studies (please go to study websites for complete instructions).

## Abbreviations

BMI: Body mass index
GWAS: Genome wide association studies
HDL: High-density lipoprotein
HRC: Haplotype Reference Consortium
HWE: Hardy Weinberg equilibrium
IV: Instrument variable
LD: Linkage disequilibrium
MR: Mendelian randomization
pQTL: Protein quantitative trait loci
