# Supplementary material for: Proteomic analysis of 92 circulating proteins and their effects in cardiometabolic diseases
Source: Clin Proteomics. 2023 Aug 7;20:31. doi: 10.1186/s12014-023-09421-0 (PMC10405520; doi:10.1186/s12014-023-09421-0)
Supplement: Supplementary file 2 — Additional file 2: Figure S1. Steps of analysis performed, as described in methods section. Figure S2. Meta-analysis methods and determination of primary and secondary pQTLs and Mendelian randomization instruments. Figure S3. Phenotypic correlation matrix across all proteins analyzed. Figure S4. Genotypic correlation matrix across all proteins analyzed. [file 12014_2023_9421_MOESM2_ESM.docx]

**Figure S1**

**
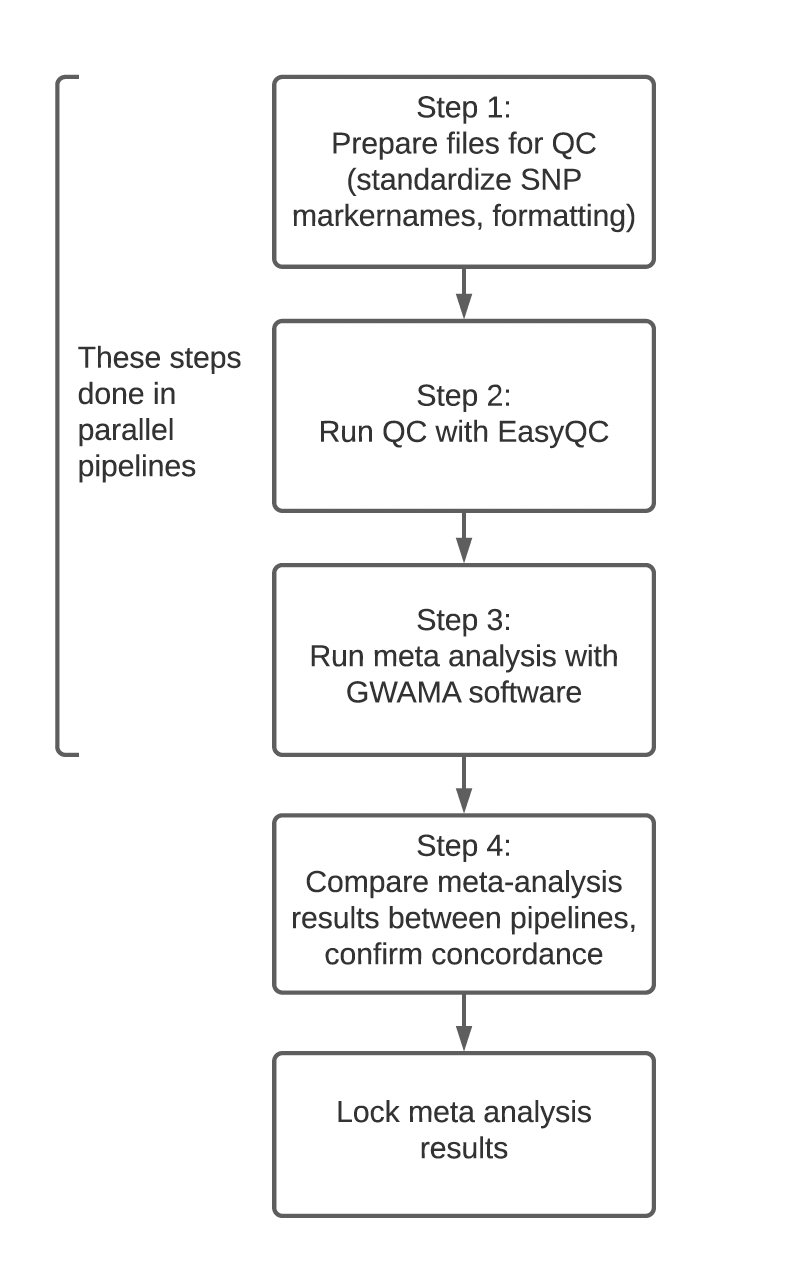
**

Steps of analysis performed, as described in methods section.

**Figure S2**

**
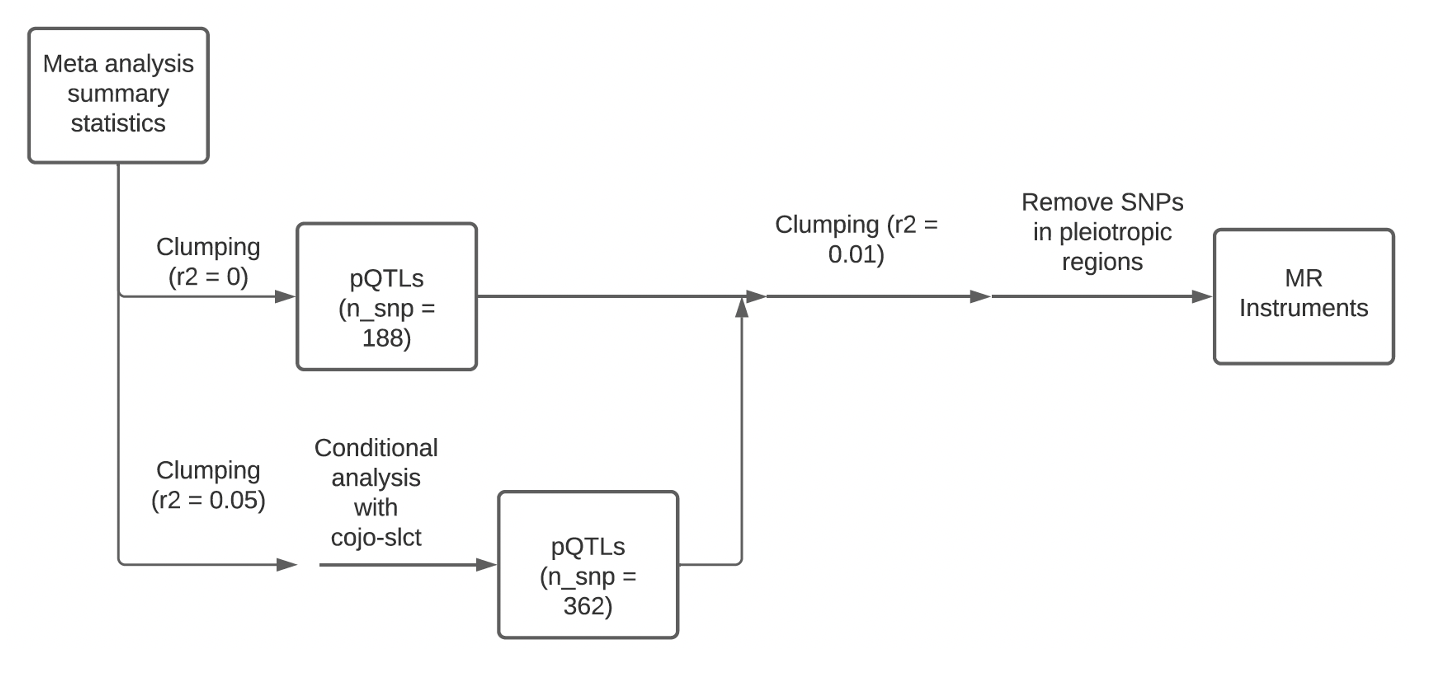
**

Meta-analysis methods and determination of primary and secondary pQTLs and Mendelian randomization instruments.

**Figure S3**


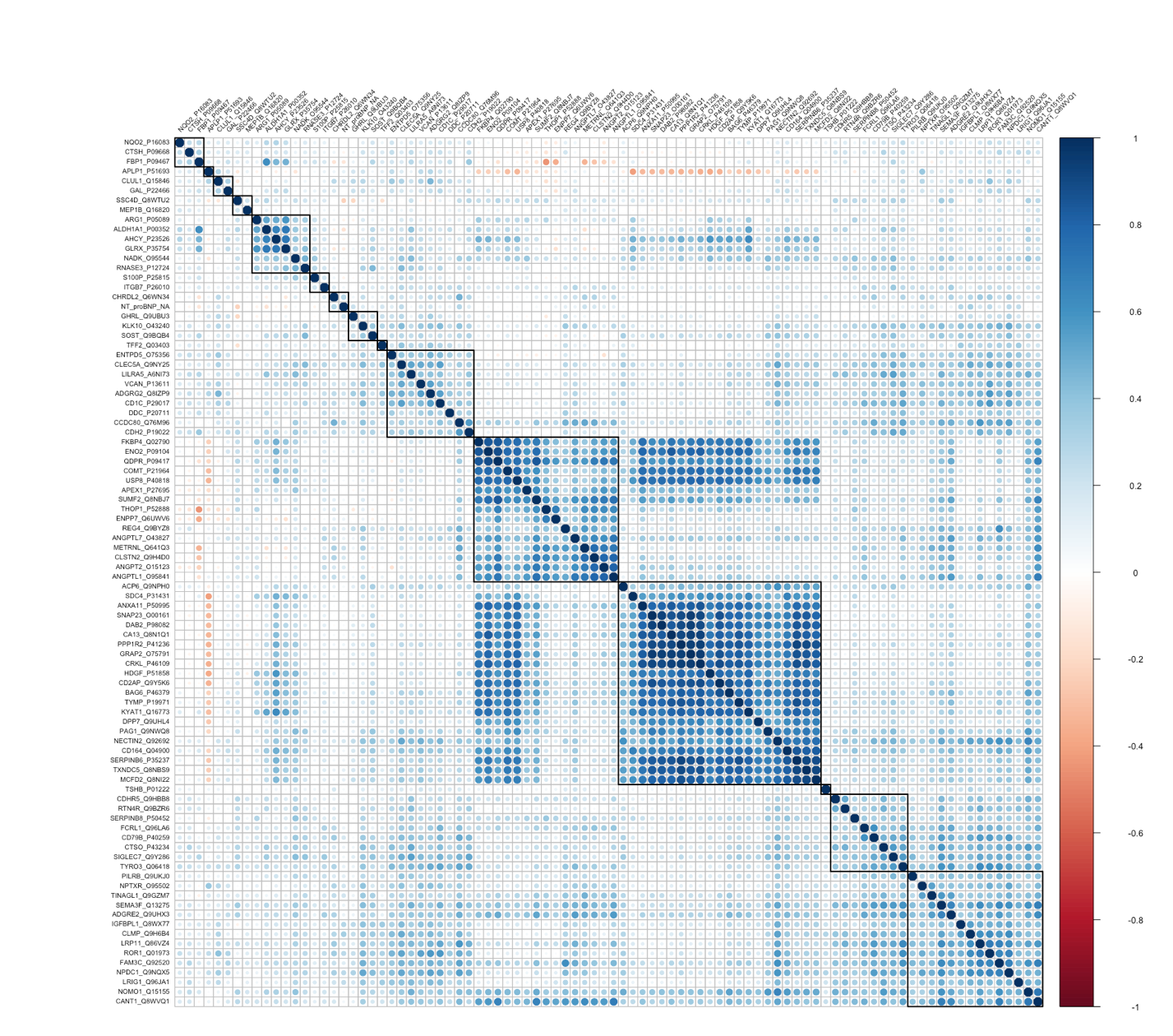


Phenotypic correlation matrix across all proteins analyzed.

**Figure S4**


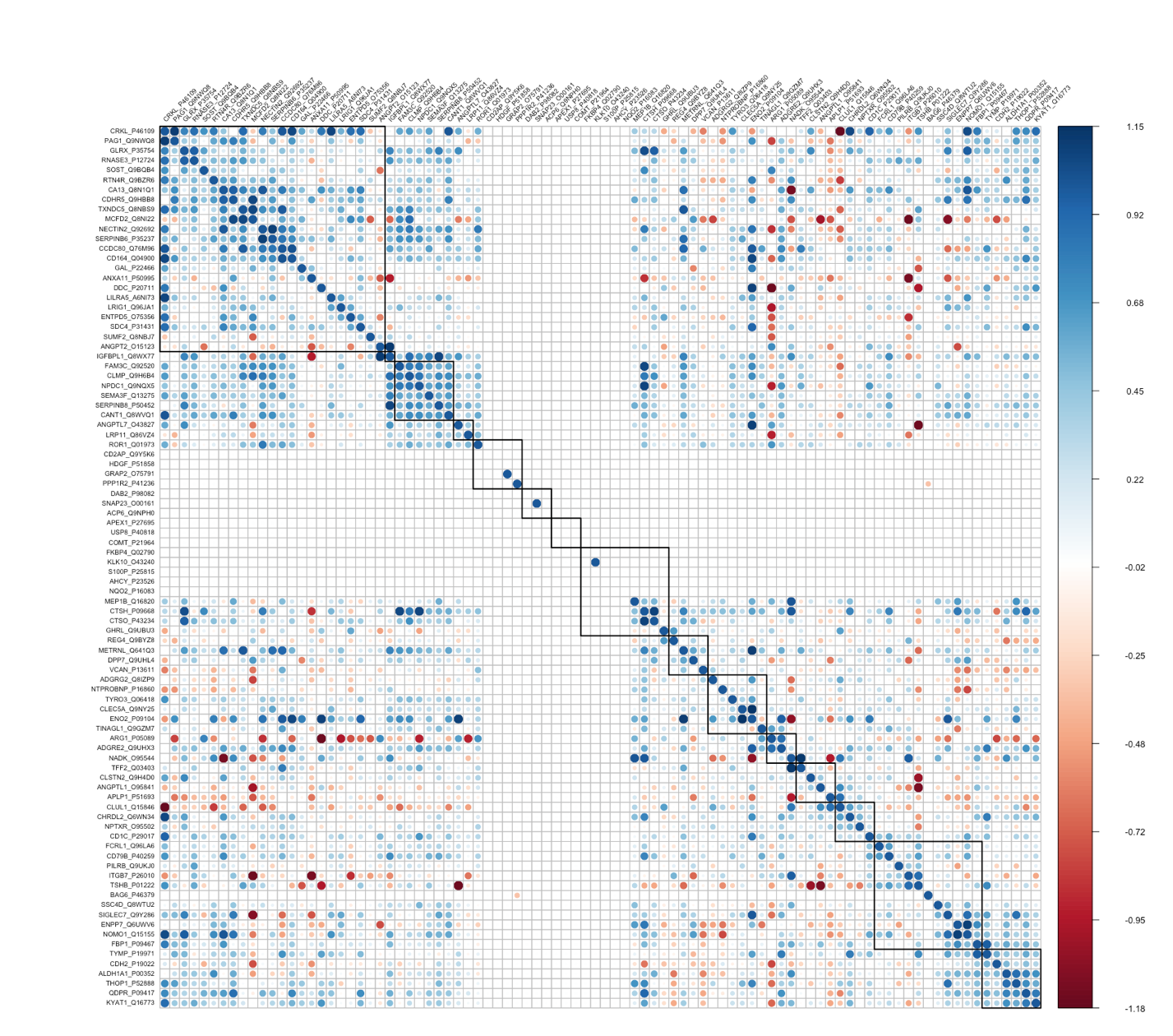


Genotypic correlation matrix across all proteins analyzed.
